# Cognitive aging in migraine sufferers is associated with more subjective complaints but similar age-related decline: a 5-year longitudinal study

**DOI:** 10.1186/s10194-020-01100-x

**Published:** 2020-04-07

**Authors:** Isabel Pavão Martins, Carolina Maruta, Pedro Nacimento Alves, Clara Loureiro, Joana Morgado, Joana Tavares, Raquel Gil-Gouveia

**Affiliations:** 1 Departamento de Neurociências e Saúde Mental, Centro Hospitalar Universitário Lisboa Norte - Hospital de Santa Maria, Lisboa, Portugal; 2 Laboratório de Estudos de Linguagem, Instituto de Medicina Molecular, Faculdade de Medicina, Universidade de Lisboa Portugal, Av Professor Egas Moniz, Lisboa, Portugal; 3 Universidade Católica Portuguesa, Católica Research Centre for Psychological - Family and Social Wellbeing, Lisboa, Portugal; 4 Serviço de Neurologia, Hospital Beatriz Angelo, Loures, Lisboa Portugal; 5 Serviço de Imagiologia, Hospital de Santa Maria, Lisboa, Portugal; 6 Headache Center, Hospital da Luz, Lisboa, Portugal

**Keywords:** Migraine headache, Cognitive performance, 5-year follow-up, Pain, Executive deficits

## Abstract

**Objectives and background:**

The effect of headache on cognitive performance is controversial, due to conflicting results obtained from studies in clinical or population settings. We aimed to understand if migraine and other headaches modify the rates of decline on different cognitive measures, during a 5-year interval.

**Design and method:**

A cohort of community dwelling adults (> 50 years) with migraine (MH), non-migraine headaches (NMH) and controls without headache (WoH), was assessed by a comprehensive neuropsychological battery with tests of memory, language and executive functions, repeated 5 years apart. Change in performance between baseline and reevaluation was compared between groups, and controlled for age, gender, literacy and depressive symptoms.

**Results:**

A total of 275 participants (78.5% WoH, 12.7% MH, 8.7% NMH) were reevaluated (average age 70.40 + 8.34 years, 64% females). Cognitive decline or dementia occurred in 11.4%, with a similar proportion among the three groups. Although MH participants had significantly more subjective cognitive complaints (*p* = 0.030, 95%CI:]-3.929,-0.014[), both MH and NMH subjects showed an age-associated decline identical to controls. Furthermore, migraine features (disease and attack duration, frequency and aura) were unrelated with cognitive performance.

**Conclusion:**

Migraine and non-migraine headache are not associated with increasing risk of dementia or cognitive decline at an older age although subjects with migraine have more cognitive complaints. Longer longitudinal studies are necessary to understand if this pattern persists for more than 5 years.

## Short summary

This study shows that older individuals with migraine and other headaches do not have an increased risk of cognitive decline, cognitive impairment or dementia than subjects without headaches, over a period of 5 years. However, subjects with migraine tend to present more subjective cognitive complaints compared to people without headaches. Longer studies are necessary to understand the impact of these complaints during aging.

## Introduction

The interaction between migraine and cognition is dynamic and seems to fluctuate along the migraine cycle. Cognitive symptoms are a very consistent feature of the attacks [1, 2] and have been substantiated by the finding of neuropsychological impairments in executive functions, memory and learning that revert to normal after the attack [3, 4]. However, the brain processes underlying these phenomena remain speculative.

Some patients also complain of cognitive changes outside the attacks, and cross-sectional controlled inter-ictal studies, in clinically based samples, have identified negligible to small effects of migraine on visuomotor processing speed, sustained attention, verbal learning and recall, prospective and working memory [5–11], that tend to be more expressive in some patient subgroups, such as migraine with aura and severe or chronic migraine [12, 13]. The finding of inter-ictal brain imaging structural and perfusion changes in migraineurs [14, 15] supported the hypothesis that migraine associated white matter abnormalities and brain lesions [16, 17] could increase the risk of late-life cognitive impairment or dementia.

However, most cross-sectional studies on population-based samples, using less detailed neuropsychological evaluations, were unable to document such changes [12, 13], although a few studies with extensive batteries and large samples identified worse [18] or even better [19] cognitive performance in migraine patients. Nevertheless, evidence obtained from large population-based longitudinal studies does not associate migraine to an increased risk of cognitive decline [20–23] nor to the progression of white matter abnormalities or infarct-like lesions [24].

This contrasting evidence stimulated the debate on whether cognitive changes identified in migraine patients, regardless of the setting, are specific to migraine and/or headache or due to confounders. In fact, executive and cognitive impairments have also been documented in other chronic or recurrent pain disorders [25]. Moreover, migraine sufferers have a higher risk of depression and anxiety and may take medication that interferes in cognitive performance. On the other hand, they may also have protective factors, such as a lower exposure to vascular risk factors due to the adoption of a healthier lifestyle in order to avoid the attacks, and/or to better disease prevention attitudes related to more frequent need to seek medical attention [26].

In a previous cross-sectional study, we compared the inter-ictal cognitive performance of adults with or without headaches or migraine, concluding that most cognitive functions and tests were not influenced by the presence of migraine or non-migraine headache in late adult life [27]. However, both subjects with migraine and non-migraine headache performed worse in a few executive tests, suggesting that persistent or recurrent pain could have some impact on executive functioning. Executive abilities are known to be particularly vulnerable to both normal cognitive ageing [28] and subcortical white matter changes [29], and therefore the question is raised on how these impairments could evolve over time.

In the current study, we aimed to determine if migraine and non-migraine headaches in adult life modify the rates or processes of cognitive aging, specifically executive functioning and memory, by comparing cognitive changes between baseline and a 5-year follow-up, between subjects with and without headaches or migraine. Our hypothesis was that there should be no difference between participants with headache and controls.

## Methods

### Participants and study design

This was a prospective longitudinal observational study on aging and cognition involving a cohort of 402 community-dwelling adult volunteers attending primary care centers of the National Health Service in the region of Lisbon. Subjects were screened and invited to participate by their general practitioner provided that, at inception, they had a minimum age of 50 years, spoke Portuguese as their native language, were autonomous for instrumental daily living activities and scored within literacy-adjusted normal values [30] on the Mini Mental State Evaluation (MMSE) [31]. Exclusion criteria included a history of any neurologic or psychiatric disease (ex. stroke, brain injury, epilepsy, dementia or psychosis) and any severe medical disorder with potential influence on neurological function (ex. cancer, HIV infection, renal or hepatic failure). Further details of the study design and baseline evaluation have been published elsewhere [27].

**Fig. 1:**
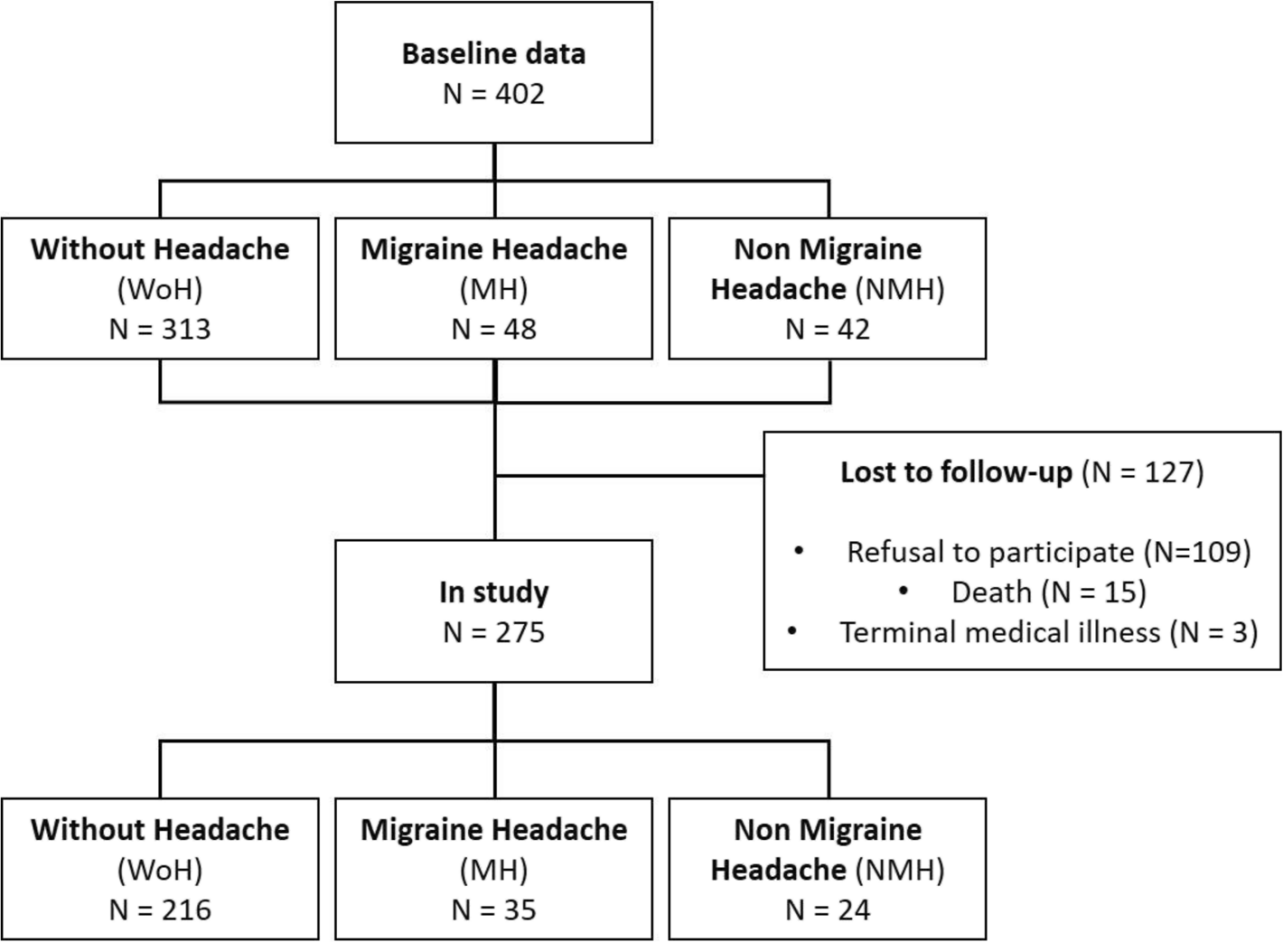
Flowchart of study participants from baseline to follow-up

The participants of this study came from a cohort taking part in a prospective cross-sectional study composed by 479 subjects that performed a baseline assessment [32]. However, 77 of those were excluded from the longitudinal follow up because they could not be reached due to both unknown contact/mailing address and insufficient updated clinical information (*N* = 53), or currently living outside the Lisbon Metropolitan region (*N* = 24). This produced a cohort of 402 participants eligible for follow-up assessment. From these, 127 were considered lost to follow-up due to refusal to participate (*N* = 109), death (*N* = 15) and terminal medical illness (*N* = 3) (see Fig. 1). This led to a final cohort of 275 subjects that participated in the follow-up part of the study after an average time interval of 4.9 (±0.6) years (Fig. 1).

The study was carried out in accordance with the recommendations of the Ethics Committee of the Lisbon Academic Medical Center (LAMC). All subjects gave written informed consent in accordance with the Declaration of Helsinki, for both parts of the study. The protocol was approved by the Ethics Committees of the LAMC, the Portuguese Health Authority and local primary care centers.

### Measures and procedures

The following data were collected on both periods of the study: (1) medical history, including vascular risk factors (hypertension, diabetes, smoking history and dyslipidemia), co-morbidities, and medication; (2) sociodemographic data (employment, cohabitation and marital status); (3) subjective cognitive complaints and depressive symptoms; and (4) cognitive performance.

Neuropsychological evaluations were performed by fully licensed neuropsychologists. It comprised the MMSE [31], tests of episodic memory [Verbal-Paired Associates, Immediate and Delayed Logical Memory and Visual Reproduction from Wechsler Memory Scale – III (WMS-III) [33]] and the 9-item version of the California Verbal Learning Test [34]), semantic memory [Vocabulary subtest from Wechsler Abbreviated Scale of Intelligence (WASI) [35]] attention/processing speed [Symbol search, Wechsler Adult Intelligence Scale-III (WAIS-III) [36]], and executive functions (Trail Making Test A and B (TMT-A an TMT-B)] [37], Stroop Test [38, 39] and Digit Span Backwards [36]). Language skills were assessed by semantic (Food and Animals) and phonemic Verbal Fluency. Individual scores were converted to age and education-adjusted z-scores according to the existing norms [40, 41].

In addition, the 15-item Geriatric Depression Scale [42], a subjective memory complaints questionnaire (SMCQ) [43] and the Instrumental Activities of Daily Living Scale (IADL) [44] were also applied.

### Cognitive status

For the purpose of summarizing cognitive data and classifying the subjects, an executive and a memory function composite score were calculated, resulting from the average standard scores obtained on five executive tests [(Ʃ Z Trail Making Test A + Z Trail Making Test B + Z Semantic Food Fluency + Z Semantic Animals Fluency + Z Phonemic Fluency)/5] and two episodic memory tests [(Ʃ Z Logical Memory + Z Verbal Paired Associates)/2], respectively. An additional global cognitive score was obtained consisting of the mean of memory and executive composite scores [(Ʃ Composite executive + Ʃ Composite memory) / 2].

Individuals were classified as cognitively impaired/dementia if they scored below − 1.5 standard deviations on any of the composite scores (dementia if they also had impaired daily living activities). This cut-off is frequently used to define mild cognitive impairment (MCI), it allows for the identification of a large number of cases qualifying for this diagnosis [45–47] and the diagnosis made using this cut-of has been significantly associated with measures of medial temporal atrophy and APOE genotype status [48]. These two diagnoses were further confirmed by an interview with their family.

The diagnosis of cognitive impairment/dementia was done in accordance to published criteria [49, 50] and has already been described in previous work [32]. The diagnosis was reached by consensus after consulting and analyzing the results obtained on memory and executive tests (memory or executive composite scores should be below - 1.5 SD), and the review of all available clinical, neuropsychological, and imaging data by a panel of two neurologists and two neuropsychologists. Whenever possible, an independent clinical evaluation was performed in a research center (not presented in study data) with a minimum interval of 6 months after study testing, to confirm the diagnosis of cognitive impairment. Participants were considered cognitively normal if they remained independent in daily living activities and scored at or above − 1.5 SD on both composite indexes.

### Migraine status

Headache status at baseline was classified as without headache (WoH) or with headache, which was further subdivided, according to the score obtained on the Portuguese version [51] of ID Migraine [52] into migraine headache (MH; if ID-Migraine score was ≥2) or non-migraine headache (NMH; ID-Migraine < 2).

Patients with MH at baseline were additionally contacted by telephone and systematically assessed for the current occurrence of headaches and details of headache history (disease duration, presence of aura, frequency and duration of attacks and headache impact, measured with HIT-6 [53]).

### MRI protocol and evaluation

Brain magnetic resonance imaging (MRI) was performed on a 3 Tesla Phillips scanner. The MRI protocol included conventional sequences: T13D TFE with sagittal, coronal and axial reconstructions; Axial PD/T2, FLAIR, T2* GRE and diffusion MR images. Brain MRIs were visually assessed by a neuroradiologist (JT), blind to migraine and cognitive diagnosis, to identify structural brain changes, namely white matter changes with the Fazekas scale [54, 55], and regional cerebral atrophy namely medial temporal atrophy (MTA) scale [56]. These measures were used as biomarkers of brain disease. Scores from both scales were subsequently dichotomized into normal or minor changes (scores 0–1 and moderate to severe changes (scores 2–3).

### Statistical analysis

Statistical analysis was performed with the SPSS package 21.0. Descriptive statistics were used for continuous variables and presented as mean ± standard deviation. Counts and frequencies were used for categorical variables. Group differences were tested using one-way Analysis of Variance (ANOVA) or t-tests, when appropriate, for continuous variables or Chi-Square (χ^2^) tests for categorical variables. Normal distribution assumption was tested by the Kolmogorov-Smirnov test. Whenever this assumption was violated nonparametric tests were used. To investigate whether the presence of headache and migraine were associated with increased risk of cognitive impairment/dementia at follow-up, a logistic regression analysis was performed after controlling for gender, age at follow-up and depressive symptoms. In order to analyze the main effects of time (baseline vs follow-up) and group (MH, NMH and who) as independent factors on cognitive performance we used a mixed-repeated measures ANOVA and post-hoc Bonferroni tests, after controlling for gender, age at baseline and depressive symptoms. An additional matched case-control analysis was performed. Migraine patients were matched for age, literacy and gender to non-migraine patients (1:2 ratio). A bivariate and a multivariate conditional logistic regression were computed using the same variables as above. Results were considered statistically significant at *p* < 0.05. Parameter uncertainty was indicated by the 95%CI. No statistical power calculation was conducted prior to the study. The sample size was based on the available data. We estimated that our study would be able to detect a medium effect.

## Results

The follow-up study population included 275 individuals. Of these, 176 (64%) were female, with an average age of 70 years (ranging between 55 and 98 years). Concerning the headache diagnosis, 216/275 (78.5%) subjects were headache-free, 35/275 (12.7%) had migraine and 24/275 (8.7%) non-migraine headache. Retention rates by group were 69% for WoH, 73% for MH and 58.5% for the NMH groups. Mean follow-up times were identical between groups (*p* = 0.909).

### Participant characteristics and imaging data by headache diagnosis

**Fig. 2:**
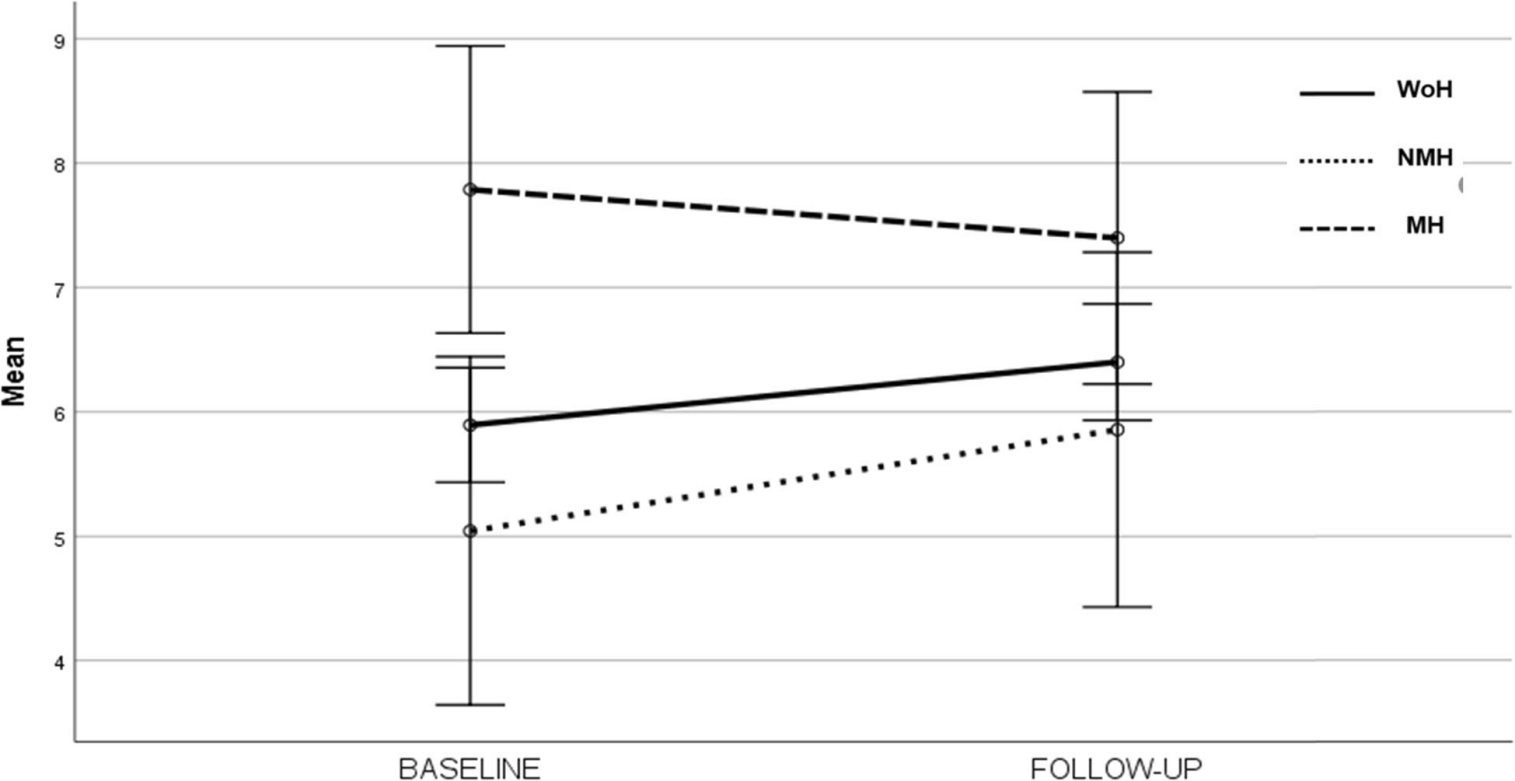
Cognitive complaints scores by headache group. *Legend:* y-axis represent estimated marginal means of Subjective Memory Complaints Questionnaire scores controlling for age and depressive symptoms; WoH – without headache, NMH – non migraine headache, MH – migraine headache

Differences between groups in demographic, imaging and clinical data are depicted in Table 1. Migraine participants were more often female, younger, had more depressive symptoms and more subjective memory complaints than WoH subjects. The number of subjective cognitive complaints was significantly different among headache groups, after controlling for age and depressive symptoms (*F* = 3.926; *p* = 0.021), being higher at baseline and remaining high and stable with aging in MH as opposed to NMH subjects (mean diff. = − 2.037, *p* = 0.030, 95%CI:]-3.929, − 0.014[) in whom they increased with age (Fig. 2). No statistical differences were found between the three groups on literacy, MMSE scores or vascular risk factors (Table 1).

**Table 1:** Population characteristics by diagnosis

	**Without headache** (WoH)	**Non migraine headache** (NMH)	**Migraine headache** (MH)	**Statistics**	***p*-value**
N	214	24	35		
Follow-up time^a^ (yrs; median [P25^th^,P75^th^])	5.1 [4.7,5.3]	5.0 [3.8,5.4]	5.2 [4.9,5.3]	W = 0.192	0.909
Gender (F:M; n(%))	126 (60%):90	17 (70%):7	33 (94%):2	χ^2^ = 17.430	**< 0.0001**
Age at baseline (yrs; mean ± sd)	65.8 ± 8.4	68.4 ± 6.8	**61.1** ± 7.4	F = 6.741	**0.001**
Age at follow-up (yrs; mean ± sd)	70.8 ± 8.5	73.0 ± 6.6	**66.1** ± 7.3	F = 6.246	**0.002**
Literacy (yrs; mean ± sd)	7.8 ± 4.2	6.5 ± 4.4	6.1 ± 4.2	F = 3.040	0.049
MMSE^a^ (median [P25^th^,P75^th^])	29 [28,30]	28 [26,29]	28 [27,29]	W = 7.143	0.028
GDS^a^ (median [P25^th^,P75^th^])	2 [1,5]	3 [2,6]	**5 [3,8]**	W = 24.019	**< 0.0001**
SMCQ (mean ± sd)	6.2 ± 3.7	6.3 ± 4.1	**8.3 ± 3.9**	F = 4.584	**0.010**
Vascular Risk Factors (0: 1: 2: ≥ 3; n)	25: 71: 144: 86	2: 10: 21: 14	4: 12: 27: 8	χ^2^ = 4.228	0.646
Cognitive decline (Yes: No; n(%))	26 (12.0%):190	3 (12.5%):21	2 (5.7%):33	χ^2^ = 1.243	0.537
*Imaging data*					
Brain MRI (Yes: No; n(%))	122 (56.5%):94	11 (45.8%):13	28 (80.0%):7	χ^2^ = 8.615	0.013
Fazekas score (0-I: II-III; n(%))	82 (68.3%):38	6 (54.5%):5	16 (57.1%):12	χ^2^ = 1.873	0.392
MTA (0–1: 2–3; n(%))	111 (90.2%):12	11 (100%):0	28 (100%):0	χ^2^ = 4.109	0.128

Figures in bold represent statistically significant differences between one group and the others after Bonferroni post hoc test

*F* females, *M* males, *MMSE* Mini Mental State Examination, *MTA* Medial Temporal Atrophy, *GDS* Geriatric Depression Scale, *SMCQ* Subjective Memory Complaints Questionnaire

*p* ≤ 0.05 was considered significant

^a^skewness <− 1 or > 1

A total of 159 of these participants had an MRI evaluation, 120 from the who group, 13 with NMH and 23 from the MH group. No significant differences were found between groups regarding either white matter changes or atrophy measures (Table 1).

### Risk of cognitive impairment/dementia

Cognitive decline/dementia was documented in 31/275(11.3%) of the reevaluated sample with similar proportions in subjects with MH (χ^2^ = 1.239, *p* = 0.537), NMH and WoH (Table 1).

After controlling for gender, age at follow-up and depressive symptoms, the presence of migraine or nonmigraine headache were not significant predictors of cognitive impairment at follow-up. Age at follow-up was a significant predictor (Table 2). The model showed adequate calibration (Hosmer Lemeshow test χ^2^ = 10.051; *p* = 0.189).

**Table 2:** Predictors of cognitive impairment (multivariable logistic regression analysis)

**Predictors**	***Wald*** χ^2^ value	***p*-value**	**OR**	**95% CI**	
				*Inferior*	*Superior*
*MH*	0.289	0.591	0.647	0.132	3.160
*NMH*	0.012	0.914	0.927	0.238	3.619
*Gender*	0.696	0.404	0.703	0.308	1.608
*Age at follow-up*	**11.929**	**0.001**	**1.088**	**1.037**	**1.141**
*GDS*	1.961	0.161	1.084	0.968	1.213

*MH* migraine headache, *NMH* non-migraine headache, *GDS* Geriatric Depression Scale, *OR* odds ratio, *CI* confidence interval

Significance is set at *p* < 0.05

In the matched case-control analysis, there was no significant association between migraine and cognitive impairment as well, both in the bivariate analysis (*p* = 1.00, OR = 1, 95% CI [0.09–11.92]) and in the multivariate analysis (*p* = 0.778, OR 1.58, 95% CI [0.08–28.64]).

### Cognitive performance between headache groups

In order to analyze the main effects of time (baseline vs follow-up) and group (MH, NMH and who) as independent factors on cognitive performance we used a mixed-repeated measures ANOVA and post-hoc Bonferroni tests, after controlling for gender, age at baseline and depressive symptoms.

**Fig. 3:**
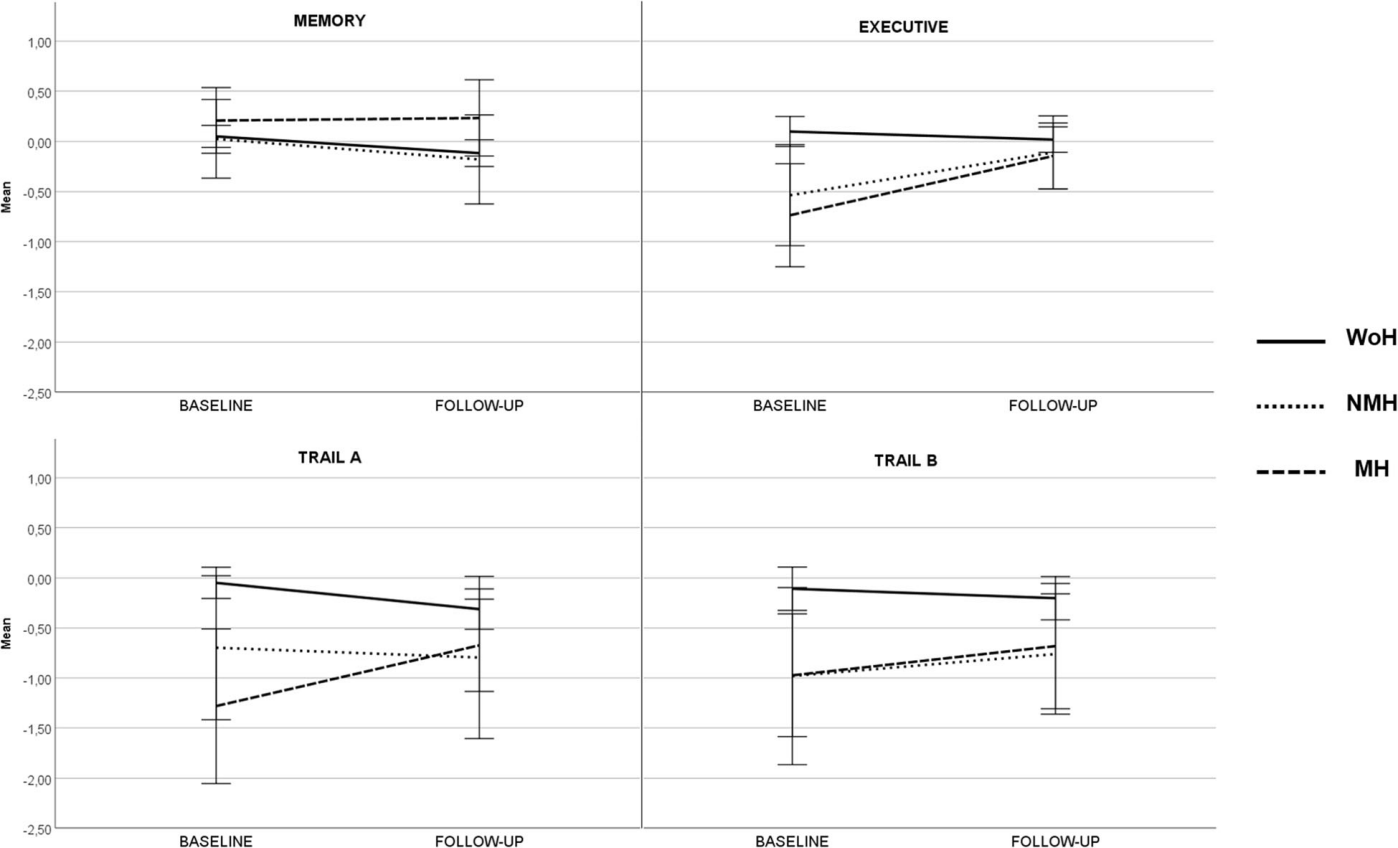
Cognitive performance overtime by headache group. *Legend:* y-axis represent estimated marginal means memory and executive composite zscores and trail A and B zscores controlling for gender, age at baseline, depressive symptoms; WoH – without headache, NMH – non migraine headache, MH – migraine headache

All groups showed a significant decline in memory, from baseline to follow-up (*F* = 17.878, *p* < 0.0001), despite a more pronounced slope of decline in WoH and NMH groups when compared to MH (Fig. 3). A significant interaction between time and age on the performace of memory composite score was also identified (*F* = 17.556, *p* < 0.0001). In addition, a significant main effect of headache type was found on the performance of executive function (*F* = 3.706, *p* = 0.026) but not on memory composite scores (*F* = 0.398, *p* = 0.672). A significant interaction between group membership and time/change was also observed for the executive composite score (*F* = 4.094, *p* = 0.018). In this case, the mean difference was higher in MH (also with an increase in performance from baseline to follow-up) when compared to WoH group (mean diff. = − 0.424, *p* = 0.028, 95%CI:]-0.815, − 0.033[). As depicted in Fig. 3, NMH also showed an increase in executive function performance over time, although not different from the other two groups. The bivariate comparisons between headache diagnosis and executive performance at baseline and at follow up are detailed in Table 3.

**Table 3:** Bivariate comparison between headache diagnosis and executive performance at baseline and at follow up

	**Executive score** **Baseline**	**Executive score** **Follow up**	***p*-value**
**Migraine headache** (MH)	−0.7 ± 1.5	−0.1 ± 1.0	0.0439
**Non migraine headache** (NMH)	−0.5 ± 1.2	−0.1 ± 0.9	0.0455
**Without Headache** (WoH)	0.1 ± 1.1	0.03 ± 0.9	0.0969
***p*-value**	0.0004	0.2247	

Looking into detail at each specific measure of the executive function composite score, a significant main effect of headache type was observed for both TMT-A (*F* = 3.773, *p* = 0.024) and TMT-B (*F* = 3.796, *p* = 0.024) scores. A significant interaction between headache type and TMT-A score change was also identified (*p* = 0.023). Mean change in TMT-A performance was significantly higher in the MH group, due to an increase in processing speed score of overtime, when compared to WoH subjects (mean diff. = − 0.579, *p* = 0.044, 95% CI:]-1.147, − 0.011[). As depicted in Fig. 2, MH was the only group where this improvement was noted, whereas the remaining groups showed a decline over time.

In order to control for group differences observed in the baseline evaluation, post-hoc analyses were repeated using the arithmetical difference between baseline and follow-up memory and executive function composite scores. Once again, a significant difference between MH (− 0.59 ± 1.5) and WoH (0.07 ± 1.0) was observed in executive (*F* = 4.090, *p* = 0.018) but not memory performance (*F* = 0.376, *p* = 0.658), although this difference did not reach statistical significance when analyzing specific cognitive tests (*p* values between 0.197 and 0.967).

### Migraine data at follow up

From the 35 MH patients included at baseline, 29 (83%) were successfully interviewed after the neuropsychological revaluation and 5/35 were impossible to reach. Nine patients out of the 35 (31%) reported having aura with their headaches, but only one patient reported it in all the attacks. All subjects had migraine for more than 10 years; disease duration was less than 20 years in 11/35, between 21 and 50 years in 7/35, and between 51 to 70 years in 5/35 cases.

Twenty patients had suffered migraine attacks in the last year, although in most, their frequency was scarce. Thirteen (65%) patients had up to one attack per month, five (25%) had more than 15 monthly headache days (chronic migraine) and two had daily headache. Average attack duration was 17.9 ± 22.0 h, ranging from 45 min to 3 days; one of the daily headache patients had continuous headache. Average HIT-6 score of this sample was 54.3 ± 11.1 (range 36 to 76). Some patients noticed a change in their migraine features at follow up, as 11/20 (55%) ceased to have nausea with their attacks, 6/20 (30%) ceased to be disturbed with photophobia and 7/20 (35%) were now able to work and function during their migraine attacks.

### Cognitive performance in migraine

In the MH group, the two patients presenting cognitive decline had aura (χ^2^ = 4.320, *p* = 0.038) but the presence of aura had no influence on the executive (t = 0.639, *p* = 0.529.) or memory scores (t = 1.052, *p* = 0.266). Disease duration (χ^2^ = 0.157, *p* = 0.541), attack frequency (t = 0.332, *p* = 0.743), attack duration (t = − 1.299, *p* = 0.210) and Fazekas score (χ^2^ = 2.128, *p* = 0.546) had no association with cognitive decline in migraine patients. Also, MH subjects showing a decline in cognitive functions had similar HIT-6 scores as those who remained cognitively normal (t = − 2.216, *p* = 0.041); the HIT-6 score did not correlate with the executive function (*r* = − 0.248, *p* = 0.307) or memory scores (*r* = − 0.246, *p* = 0.269).

## Discussion

In this study, we did not find evidence for a) increased risk of cognitive decline or dementia or b) steeper cognitive decline, over a 5-year period, in subjects with migraine and non-migraine headaches, when compared to individuals without headache. Nonetheless, we found that individuals with migraine presented more subjective cognitive complaints and depressive symptoms than controls.

The present study included participants mainly between the 5th and the 7th decades of life and corroborated previous evidence from population-based longitudinal studies [20–24]_,_ using a variety of cognitive measures and with follow-up times ranging between 6 and 12 years, showing that persisting migraine in older adults does not increase the risk of cognitive decline and does not influence performance in memory and executive tests. Although the follow-up time can be considered relatively short, similar intervals were sufficient to document age-related cognitive decline in population studies. The effect of non-migraine headache on cognition has only been studied in the Epidemiology of Vascular Aging (EVA) study [22] and, similarly to our results, also produced negative findings regarding cognitive performance, despite the fact that subjects with headaches, particularly migraine with aura, had more white matter hyperintensities than controls [57]. The consistency of these results is reassuring and it seems reasonable to assume that persisting headache and/or migraine at old age does not increase the risk of cognitive decline.

In general, population-based cross-sectional controlled studies on cognition in migraine have also been negative [12, 58], with two exceptions. In one of them, 20% of the participants suffered from chronic migraine, a disorder known for having a high impact on subjects’ lives and several associated co-morbidities that may have biased the results [18]. The other exception was the cross-sectional baseline analysis of our own population that revealed lower performance in both MH and NMH subjects on a few executive measures. Interestingly, both exceptions included participants with low literacy and it is well known that education is an important determinant of cognitive performance. A large cross-sectional study, in which about half of the participants had medium to higher education, produced the opposite trend, i.e. of a better cognition in migraine subjects [19].

Our study also showed that cognitive decline in migraine patients was neither associated with measures of migraine activity, intensity or impact nor with brain biomarkers (Fazekas scores and cortical atrophy). The lack of association between white matter lesion load and poorer neuropsychological performance has been previously described [6] and it can be subject to confounders (e.g., cardio- and cerebrovascular risk factors, namely hypertension). Moreover a longitudinal imaging study failed to show any progression of these brain lesions [24] in migraine. Likewise, migraine aura was unrelated to cognitive change, either in our sample or in other longitudinal studies [20, 23], although data from cross-sectional studies has produced conflicting findings [6, 13]. In our sample, the total number of migraine patients with cognitive decline was small (2 in total), limiting speculation about the effect of migraine features on decline.

Two additional findings emerged from this study. One was the different rate of cognitive change with time between migraine subjects and controls, in particular regarding executive function tests. The other was the consistently high rates of subjective cognitive complaints in the migraine group, was found both at baseline and follow-up.

Although most measures (Table 4) showed a tendency to decline with age, individuals with migraine showed less decline or even improvement with age in measures of processing speed and attention. Interestingly, MH participants also presented lower baseline scores on those tests compared to controls. A very similar pattern was described in the Baltimore Ageing study [20], especially in migraine subjects older than 50 years, who showed less decline than controls. Since the participants of the present study had lower scores at baseline, we repeated the analysis using the “follow-up minus baseline difference” as the dependent variable and obtained identical results. In both studies subjects with migraine were, on average, younger than controls, and therefore this may represent the relative sparing effect of younger age in age-related cognitive decline. Another explanation is that performance in migraineurs may be more variable over time, due to cyclic changes in brain excitability [59] leading to fluctuations in performance that regress to the mean with test repetition. The finding that migraineurs present variable results in different studies and contexts supports this hypothesis. The fact that these fluctuations are more evident in executive tests is not clear but may be associated with fluctuating attention [60]. Finally, one may not exclude the possibility that this late cognitive improvement is related to the decline of migraine activity with aging, leading to a “return to normal” of intermittent brain dysfunction. Our study was not designed to evaluate the effect of disease activity, but of disease trait. Close monitoring of migraine and cognitive functions will be necessary to disentangle this hypothesis.

**Table 4:** Mixed-repeated measures ANCOVA to analyse the main effects of change (within subjects condition) x headache diagnosis (between subjects condition), after controlling for possible confounders (age, gender and depressive symptoms)

	**WoH**		**NMH**		**MH**		**Mixed-repeated measures ANCOVA**											
	*B*	*FU*	*B*	*FU*	*B*	*FU*												
							**Change**^**a**^		**Head. Diag.**^**b**^		**Change x Head. Diag.**		**Change x gender**		**Change x age**		**Change x GDS**	
							**F**	***P***	**F**	***p***	**F**	***p***	**F**	***p***	**F**	***p***	**F**	***p***
**EXECUTIVE SCORE**	0.1 ± 1.1	0.03 ± 0.9	−0.5 ± 1.2	−0.1 ± 0.9	−0.7 ± 1.5	− 0.1 ± 1.0	0.301	0.584	**3.706**	**0.026**	**4.094**	**0.018**	1.068	0.302	0.182	0.670	0.815	0.367
*Food Fluency*	0.8 ± 1.5	0.7 ± 1.6	0.6 ± 1.6	0.5 ± 1.8	0.3 ± 1.6	0.5 ± 1.5	**4.532**	**0.034**	2.500	0.084	0.053	0.948	1.148	0.285	**5.921**	**0.016**	0.006	0.936
*Animals Fluency*	0.2 ± 1.0	0.1 ± 1.1	0.3 ± 1.1	0.2 ± 0.8	0.2 ± 0.9	0.1 ± 0.9	0.005	0.946	0.282	0.755	0.032	0.968	0.354	0.552	0.190	0.663	0.050	0.823
*Letter “P” Fluency*	−0.2 ± 1.0	−0.1 ± 1.0	0.2 ± 1.5	0.1 ± 0.9	−0.01 ± 1.0	− 0.4 ± 0.9	0.000	0.996	2.098	0.125	1.645	0.195	0.795	0.373	0.002	0.961	**6.787**	**0.010**
*TMT A*	−0.04 ± 1.1	− 0.3 ± 1.5	− 0.7 ± 1.7	−0.9 ± 2.0	−1.3 ± 2.1	− 0.7 ± 1.2	3.114	0.079	**3.773**	**0.024**	**3.845**	**0.023**	0.006	0.937	2.994	0.085	0.235	0.628
*TMT B*	−0.1 ± 1.5	−0.2 ± 1.5	−1.0 ± 1.7	− 0.8 ± 1.1	−1.0 ± 1.5	−0.7 ± 1.5	3.505	0.063	**3.796**	**0.024**	1.314	0.271	0.020	0.888	2.190	0.140	6.449	0.012
**MEMORY SCORE**	0.1 ± 0.8	−0.1 ± 1.0	0.01 ± 0.9	−0.2 ± 1.1	0.2 ± 0.9	0.2 ± 1.1	**17.877**	**< 0.00001**	0.398	0.672	0.372	0.690	1.719	0.191	**17.556**	**< 0.00001**	1.646	0.201

Figures in the descriptive statistics part of the table represent zscores

*B* baseline, *FU* follow-up, *MH* migraine headache, *NMH* non migraine headache, *TMT* Trail Making Test, *WoH* without headache

Significance set at *p* < 0.05

^a^within-subjects condition

^b^between-subject condition

The other finding is the association between migraine and subjective cognitive complaints, a very consistent finding between baseline and follow-up, in the absence of decline. Cognitive complaints are common in headache practice and are known to correlate with depressive symptoms yet, in this study, their number remained high after controlling for depressive symptoms.

Finally, it is worth mentioning that migraine features changed over the timespan of 5 years. In this sample, 31% of migraine patients who were reevaluated had no attacks in the previous year and 65% of those with persistent attacks had less than one attack per month. In 55% of subjects, attacks ceased to be accompanied by nausea, in 30% the photophobia was no longer present and 35% had milder attack impact, being now able to work and function during attacks. These changes in migraine characteristics with aging have been documented [61, 62], but clearly influence our ability to diagnose migraine and to distinguish it from non-migraine headache, being a recognized limitation that may influence the results of cross-sectional studies that rely on a single evaluation of older individuals.

We acknowledge some limitations in this study. One is the high attrition rate (around 32%), when compared to similar length studies that had retention rates of 80% at 3 years [23], 98% at 5 [22] and 75% at 6 years [21] follow-up. Possible explanations may include the lack of financial compensation for participation, higher age and lower population education levels. Also, there were some demographic differences between groups, since migraine sufferers were younger, more often females and had higher depression rates and subjective memory complaints, although their cognitive performance was uninfluenced by these factors [27]. Thirdly, the number of participants with headache or migraine was small, and the distinction between MH and NMH was not based on a clinical assessment, but on a reliable instrument (ID-Migraine), without further specifying the headache subtypes in the NMH group. Finally, we did not calculate the power or the sample size necessary to obtain differences between groups. High-powered studies in the future are needed to draw more definitive conclusions.

The strong aspects of this study are the detailed cognitive assessment and the confirmation of migraine diagnosis at follow-up.

## Conclusion

Although persisting migraine and non-migraine headache may influence some measures of executive performance these headaches are not associated with an increased risk of cognitive decline, suggesting that repeated migraine attacks do not have a long-term impact on cognition. However patients with migraine tend to report more subjective cognitive complaints during aging. Longer follow-up studies are necessary to corroborate these results.

## Data Availability

The datasets for this manuscript are not publicly available because Datasets belong to Language Research Laboratory (Faculty of Medicine of Lisbon) and are currently still being analyzed. Requests to access the datasets should be directed to isabel_martins@medicina.ulisboa.pt.

## Abbreviations

ANOVA: Analysis of variance
EVA: Epidemiology of Vascular Aging
IADL: Instrumental Activities of Daily Living
LAMC: Lisbon Academic Medical Center
MRI: Magnetic resonance imaging
MTA: Medial temporal atrophy
MH: Migraine headache
MCI: Mild cognitive impairment
MMSE: Mini-Mental State Examination
NMH: Non-migraine headache
SMCQ: Subjective memory complaints questionnaire
TMT-A: Trail Making Test A
TMT-B: Trail Making Test B
WAIS-III: Wechsler Adult Intelligence Scale-III
WASI: Wechsler Abbreviated Scale of Intelligence
WMS-III: Wechsler Memory Scale – III
WoH: Without headache
